# Effectiveness and safety of repeated photodynamic therapy in recurrent central serous chorioretinopathy

**DOI:** 10.1111/aos.17511

**Published:** 2025-05-02

**Authors:** F. M. van den Tillaart, A. Komrojan, C. B. Hoyng, J. P. Martinez Ciriano, S. Yzer

**Affiliations:** ^1^ Department of Ophthalmology Radboud University Medical Center Nijmegen The Netherlands; ^2^ Rotterdam Ophthalmic Institute Rotterdam Eye Hospital Rotterdam The Netherlands

**Keywords:** central serous chorioretinopathy, multimodal imaging, photodynamic therapy, recurrence, repeated photodynamic therapy, subretinal fluid

## Abstract

**Purpose:**

To evaluate anatomical and functional outcomes after a second photodynamic therapy (PDT) in patients with central serous chorioretinopathy (CSC), who had a recurrence of subretinal fluid (SRF) after a previously successful PDT.

**Methods:**

In this multicentre retrospective cohort study, we included patients with CSC who underwent a second PDT because of a recurrence of SRF after an effective first PDT with complete SRF resolution. Diagnosis of CSC was based on multimodal imaging. We evaluated the complete resolution rate of SRF after a second PDT with reduced settings. Also, visual acuity (VA), subfoveal ellipsoid zone (EZ) and subfoveal external limiting membrane (ELM) integrity before and after the first and the second PDT were studied.

**Results:**

A total of 88 patients were studied, of whom 18% were female. The mean age was 51.3 ± 10.5 years at the second PDT. At the first follow‐up visit after the second PDT (median of 44 days [IQR 40–51] after PDT), a complete resolution of SRF occurred in 64% of eyes. The median VA was 0.15 LogMAR before the second PDT and improved to 0.05 LogMAR (*p* < 0.001) after PDT. The EZ was continuous in 3% of patients before the second PDT, and EZ continuity increased to 27% at the first follow‐up visit after PDT. No new atrophy was observed after the second PDT.

**Conclusion:**

A second PDT with reduced settings in recurrent CSC is effective and safe in the short term, as significant anatomical and functional improvement was observed.

## 1 INTRODUCTION

Central serous chorioretinopathy (CSC) is a chorioretinal disease that predominantly affects men between the ages of 20 and 60 years (Kido et al., 2022; Kitzmann et al., 2008). It is characterised by subretinal fluid (SRF) accumulation underneath the retina. Although the pathogenesis of CSC is not completely understood, it is believed that choroidal venous overload causes choroidal hyperpermeability and attenuation of the choriocapillaris, secondary to the dilation of choroidal vessels in Haller's layer (pachyvessels). This subsequently leads to dysfunction of the retinal pigment epithelium (RPE) and to SRF accumulation (Cheung et al., 2024; Spaide et al., 2022). Prolonged accumulation of SRF can induce irreversible changes in the RPE and photoreceptors, resulting in a loss of visual acuity (VA), and a loss of contrast and colour vision, which ultimately leads to a decrease in vision‐related quality of life (Breukink et al., 2017; van Rijssen et al., 2019).

Resolution of SRF is therefore warranted, and treatment is indicated if spontaneous resolution does not occur within 2–6 months. Photodynamic therapy (PDT) with reduced settings is the treatment of first choice (Feenstra et al., 2024; van Rijssen et al., 2019). PDT with reduced settings (i.e. half‐dose PDT, half‐time PDT or half‐fluence PDT) has been shown to be as effective as conventional PDT, while minimizing deleterious effects on the retina and choriocapillaris (Shin et al., 2011). Other treatments for CSC are subthreshold micropulse laser, oral eplerenone and laser photocoagulation. Previous randomized controlled trials observed a significantly higher SRF resolution rate after PDT when compared to subthreshold micropulse laser treatment and oral eplerenone (van Dijk et al., 2018; van Rijssen et al., 2022).

These randomised controlled trials only included treatment‐naïve patients, yet recurrence of SRF after a previous successful PDT can occur. Previous studies reported an SRF recurrence rate of 6%–25% during a mean follow‐up of 12–58 months after PDT with reduced settings (Fujita et al., 2015; Lai et al., 2015, 2016; Park et al., 2019; van Rijssen et al., 2021). Subsequently, retreatment may be warranted in the event of SRF recurrence after successful PDT (i.e. complete resolution of SRF). Given the previous effectiveness of PDT, the preferred approach is usually to repeat PDT. To date, no studies have reported on the safety and effectiveness of a second PDT in patients who had a recurrence of SRF after a previously successful PDT.

Therefore, in this study, we aim to assess the effectiveness and safety of a second PDT in patients who had a recurrence of SRF after confirmed complete resolution of SRF following the first PDT. In addition, we compare imaging characteristics between the first and the second PDT to examine disease presentation and disease progression over time.

## 2 METHODS

### 2.1 Study design

For this multicentre retrospective cohort study, data were collected from patients who underwent a second PDT due to a recurrence of SRF caused by CSC. Medical records were screened from the time PDTs were first registered at the hospitals until 2024. In the Radboud University Medical Center (Nijmegen, the Netherlands), medical records were reviewed from 1‐11‐2010 to 01‐03‐2024, and in the Rotterdam Eye Hospital (Rotterdam, the Netherlands), records were reviewed from 01‐01‐2007 to 01‐01‐2024.

This study adhered to the tenets of the Declaration of Helsinki. Due to the retrospective nature of the study, the Institutional Review Board (METC Oost‐Nederland) waived the need to obtain written informed consent.

### 2.2 Inclusion and exclusion criteria

Patients with CSC were included based on the inclusion criteria of a previously published randomized controlled trial and the previously published CSC classification (Chhablani & Cohen, 2020; van Dijk et al., 2018). According to the published CSC classification, the criteria to diagnose CSC included the mandatory presence of RPE alterations (on fundus autofluorescence (FAF), infrared imaging or optical coherence tomography (OCT)) and SRF (on OCT). In addition, at least one of the following criteria had to be met: hypercyanescence on indocyanine green angiography (ICGA); focal leakage points on fluorescein angiography (FA); or subfoveal choroidal thickness (SFCT) of at least 400 μm (Chhablani & Cohen, 2020).

In addition to the diagnosis of CSC, other inclusion criteria were: complete resolution of SRF within 6 months after the first PDT; a second PDT because of recurrence of subfoveal SRF (defined as a recurrence of subfoveal SRF, regardless of whether the location of the leakage differs from that of the previous SRF episode); and at least one follow‐up visit after the second PDT. Patients were excluded if a subthreshold micropulse laser or laser photocoagulation had been performed within 6 months before one of the PDT sessions and if patients had medicinal treatment for CSC (including MR‐antagonists, carbonic anhydrase inhibitors, etc.) within 6 weeks before PDT. Furthermore, we excluded patients if subfoveal SRF was absent at the first or the second PDT, in case of the presence of other retinal diseases that can lead to SRF, and if a secondary choroidal neovascularisation (CNV) was present. The presence of a CNV was mainly evaluated on OCT‐angiography (OCTA). In patients without OCTA imaging, the diagnosis of a CNV was made if a well‐demarcated hyperfluorescent plaque on ICGA and/or a mid‐reflective to hyperreflective flat irregular pigment epithelial detachment was present.

### 2.3 Data collection

Data were collected from medical records (VA, patient demographics, ophthalmic history, onset of subjective visual symptoms and date of first objectified SRF on OCT) and from imaging modalities including OCT, OCTA, FA, FAF and ICGA. OCT(A) imaging was performed on the Spectralis HRA + OCT device (Heidelberg Engineering, Heidelberg, Germany), Cirrus HD‐OCT (Carl Zeiss Meditec AG, Jena, Germany), OCT‐HS100 (Canon Inc., Tokyo, Japan), OCT3 Stratus (Carl Zeiss Meditec, Inc., Dublin, CA, USA) or RTVue (Optovue Inc., CA, USA). FA, FAF and ICGA were made on the Spectralis HRA + OCT device (Heidelberg Engineering, Heidelberg, Germany), TRC‐50DX (Topcon corp. Tokyo, Japan) or Zeiss FF450plus Fundus Camera (Carl Zeiss Meditec AG Jena, Germany).

### 2.4 Photodynamic therapy

PDT with verteporfin (Visudyne; Alcami Carolinas Corporation, Charleston, SC, USA) was performed with reduced settings. Half‐time PDT (6 mg/m^2^ verteporfin, 41–42 s and 50 J/cm^2^), reduced‐time PDT (6 mg/m^2^ verteporfin, 63 s and 50 J/cm^2^) or half‐dose PDT (3 mg/m^2^ verteporfin, 83 s and 50 J/cm^2^) was performed according to the preference of the treating physician. The verteporfin was activated with the standard PDT laser wavelength of 689 nm. Treatment areas were defined by the treating physician based on hyperfluorescence on FA or hypercyanescence on ICGA.

### 2.5 Outcome measures

The primary outcome measure was the effectivity of a second PDT, defined as a complete resolution of SRF. Secondary outcome measures were duration of treatment‐free interval, VA and characteristics on OCT, FA, ICGA and FAF. In both hospitals involved in this study, two follow‐up visits were routinely scheduled after PDT, which is consistent with the regimen in a previous randomised controlled trial (van Dijk et al., 2018). Therefore, we were able to study VA and OCT parameters at the visit before both PDTs, and at the first and the second follow‐up visits after the PDTs. FA, ICGA and FAF parameters were examined only prior to both PDTs, as these imaging modalities were only performed before the PDTs.

The following characteristics were evaluated on OCT: central foveal thickness (CFT, defined as the distance between the internal limiting membrane and the inner border of the ellipsoid zone (EZ) in the foveal dip); foveal external limiting membrane (ELM) integrity and foveal EZ integrity (defined as continuous/regular or irregular/indiscernible) and the presence/absence of increased or new RPE atrophy after PDT (increase of ‘comet tail’ sign) at OCT B‐scans through the fovea (Feenstra et al., 2023). FA, ICGA and FAF imaging were acquired only before the first and the second PDT. The following imaging characteristics were compared between the two time points: presence and area of diffuse atrophic RPE alterations (DARA) on FA and/or FAF; area of ICGA hypercyanescence and leakage point location on FA. All measurements and gradings were performed by two independent graders (F.v.d.T. and A.K.) and a third retina specialist was involved in cases of disagreement (S.Y.).

### 2.6 Statistical analysis

Statistical analysis was performed using SPSS Statistics (IBM corp. version 29.0 Armok, New York, USA). VA was measured in Snellen and was converted to a logarithm of the minimum angle of resolution (LogMAR) VA with LogMAR = −log(Snellen fraction). Intergrader agreement was analysed using Intraclass Correlation Coefficients (ICC) and Cohen's Kappa (Tables [Link], [Link]). For continuous variables, the paired *t*‐test was used for normally distributed outcomes, and for the related samples, the Wilcoxon signed‐rank test for non‐normally distributed outcomes. For categorical variables, the McNemar test was performed. Statistical analyses were performed on non‐missing data. A *p*‐value of <0.05 was considered statistically significant. No correction for multiple testing was performed for the part of the study in which the characteristics between the first and the second PDT were compared due to the exploratory setting.

## 3 RESULTS

A second PDT due to the recurrence of SRF after one previously performed PDT was administered to 153 patients, of whom 88 eyes from 88 patients met the eligibility criteria (Figure S1). The mean age at the second PDT was 51.3 ± 10.5 years and 18% of the patients were female (Table 1). In 39% of cases, the second PDT was performed within 2 years after the first PDT, and in 28%, the second PDT was performed after more than 5 years (Figure S2). The median duration between the start of the CSC episode (date of onset of visual symptoms or date of first objectified SRF on OCT) and the performed PDT was 4 (3–9) months for the first PDT and 3 (2–5) months for the second PDT. Eleven percent of patients had the second PDT within 6 weeks after the onset of the episode. The follow‐ups after the first PDT were at a median of 46 days (IQR 41–53) and 147 days (IQR 102–182). The first and the second follow‐ups after the second PDT were at a median of 44 days (IQR 40–51) and 94 days (IQR 84–142).

**TABLE 1:** Baseline characteristics at the first and the second PDT.

Characteristics	First PDT	Second PDT
	(*n* = 88)	(*n* = 88)
Age, Mean (SD), years	47.7 ± 10.6	51.3 ± 10.5
Female, no. (%)	16 (18%)	
Current steroid use		
None	75 (85%)	76 (86%)
Topical	4 (4%)	4 (4%)
Systemic	9 (10%)	8 (9%)
Phakic	83 (94%)	83 (94%)
PDT settings		
Half‐dose PDT	15 (17%)	24 (27%)
Half‐time PDT	73 (83%)	62 (70%)
Three‐fourth‐time PDT	0 (0%)	2 (2.2%)
Treatments before 1st PDT		
HSML	1 (1%)	N/A
MR‐antagonists	0 (0%)	
Anti‐VEGF	5 (6%)	
Laser photocoagulation	2 (2%)	
Treatment naïve	80 (91%)	
Treatments between 1st and 2nd PDT		
HSML	N/A	1 (1%)
Eplerenone		1 (1%)
Anti‐VEGF		0 (0%)
Laser photocoagulation		0 (0%)
No treatment between 1st and 2nd PDT		86 (98%)

*Note*: Data are presented as mean ± SD or as absolute numbers and percentages.

### 3.1 Resolution after the second PDT

Complete resolution after the second PDT was seen in 56/88 patients (64%) at the first follow‐up and in 47/79 patients (60%) at the second follow‐up (Figure S3). Eight of the 32 patients (25%) without SRF resolution at the first follow‐up had SRF resolution at the second follow‐up. Nine of the 56 patients (16%) with a resolution after the first follow‐up showed the presence of SRF at the second follow‐up. Nine patients had no second follow‐up visit within 1 year after PDT (second follow‐up planned, but cancelled by the patient (*n* = 4), and the second follow‐up not planned due to complete resolution (*n* = 5)). Intraretinal fluid (IRF) was present in 7/88 eyes (8%) before the second PDT. After the second PDT, IRF was present in 3 out of the 7 eyes and in 2 out of the 7 eyes at the first and the second follow‐ups, respectively.

### 3.2 Functional outcome after the second PDT

VA improved significantly after the second PDT (0.15 [IQR 0.05–0.30] LogMAR before PDT to 0.05 [IQR 0.00–0.22] LogMAR at the first follow‐up, *p* < 0.001) (Table 2, Table S2). VA decreased by two or more lines in four patients at the first follow‐up; in three of these patients, VA recovered during follow‐up. In one patient, VA did not improve; this patient had an increase of SRF after the PDT. Complete resolution in this patient occurred between 6 and 11 months after the PDT, and VA had not improved at the last follow‐up, which was 11 months after the second PDT.

**TABLE 2:** Functional and anatomical characteristics of patients with central serous chorioretinopathy at the first and the second PDT with reduced settings.

Characteristics	First PDT	Second PDT	*p*‐Value
	*n* = 88	*n* = 88	
Visual acuity, Median [IQR], logMAR			
Before PDT	0.15 [0.05–0.30]	0.15 [0.05–0.30]	0.138^a^
First follow‐up	0.05 [0.00–0.15]	0.05 [0.00–0.22]	0.174^a^
OCT			
CFT (μm), Mean (SD)			
Before PDT	112.5 ± 24.4 (*n* = 40)	105.5 ± 22.0 (*n* = 65)	0.135^b^
First follow‐up	119.8 ± 22.9 (*n* = 44)	109.7 ± 24.1 (*n* = 65)	0.052^b^
ELM integrity at fovea			
Before PDT			0.146^c^
Continuous/regular	13/40 (33%)	29/64 (45%)	
Irregular	27/40 (68%)	35/64 (55%)	
ELM integrity at fovea			
First follow‐up			>0.99^c^
Continuous/regular	24/43 (56%)	40/64 (63%)	
Irregular	19/43 (44%)	24/64 (38%)	
EZ integrity at fovea			
Before PDT			>0.99^c^
Continuous/regular	0/40 (0%)	2/64 (3%)	
Irregular	40/40 (100%)	62/64 (97%)	
EZ integrity at fovea			
First follow‐up			0.267^c^
Continuous/regular	15/43 (35%)	17/64 (27%)	
Irregular	28/43 (65%)	47/64 (73%)	
FA/FAF			
DARA			<0.001^a^
DARA <1 disc diameter	48/85 (56%)	32/83 (39%)	
DARA between 1 and 5 disc diameters	30/85 (35%)	44/83 (53%)	
DARA >5 disc diameter	7/85 (8%)	7/83 (8%)	
FA			
Leakage			0.064^c^
Focal leakage	36/83 (43%)	24/82 (29%)	
Multifocal/diffuse leakage	47/83 (57%)	58/82 (71%)	
ICGA			
Hyperpermeability			>0.99^c^
Focal hyperpermeability	7/40 (18%)	10/63 (16%)	
Multifocal/diffuse hyperpermeability	33/40 (83%)	53/63 (84%)	

*Note*: Outcome measures are presented in mean ± SD. Median [IQR] or as absolute numbers and percentages. Percentages were calculated using non‐missing data.

Abbreviations: CFT, central foveal thickness; DARA, diffuse atrophic RPE alterations; ELM, external limiting membrane; EZ, ellipsoid zone; FA, fluorescein angiography; FAF, fundus autofluorescence; ICGA, indocyanine green angiography; LogMAR, logarithm of the minimum angle of resolution; PDT, photodynamic therapy.

^a^ Related samples Wilcoxon signed rank test.

^b^ Paired *t*‐test (two‐sided).

^c^ McNemar test.

### 3.3 Anatomical outcomes after the second PDT

The CFT was 105.5 ± 22.0 μm before PDT and increased to 109.7 ± 24.1 μm at the first follow‐up (*p* = 0.043) and to 115.9 ± 26.3 μm at the second follow‐up (*p* < 0.001). The foveal ELM was continuous in 45% of patients before the second PDT, increasing to 63% at the first follow‐up (*p* = 0.078) and 70% at the second follow‐up (*p* = 0.017). Continuity of foveal EZ was observed in 3% of patients before the second PDT, and EZ restored to a continuous state in 27% of patients at the first follow‐up (*p* < 0.001) and in 37% of patients at the second follow‐up (*p* < 0.001) (Table 2, Table S2). An increase in RPE atrophy or new RPE atrophy was not observed on b‐scans centred on the fovea in any of the patients at the follow‐ups after the second PDT.

### 3.4 Comparison of visual acuity and multimodal imaging between the first and the second PDT

**FIGURE 1:**
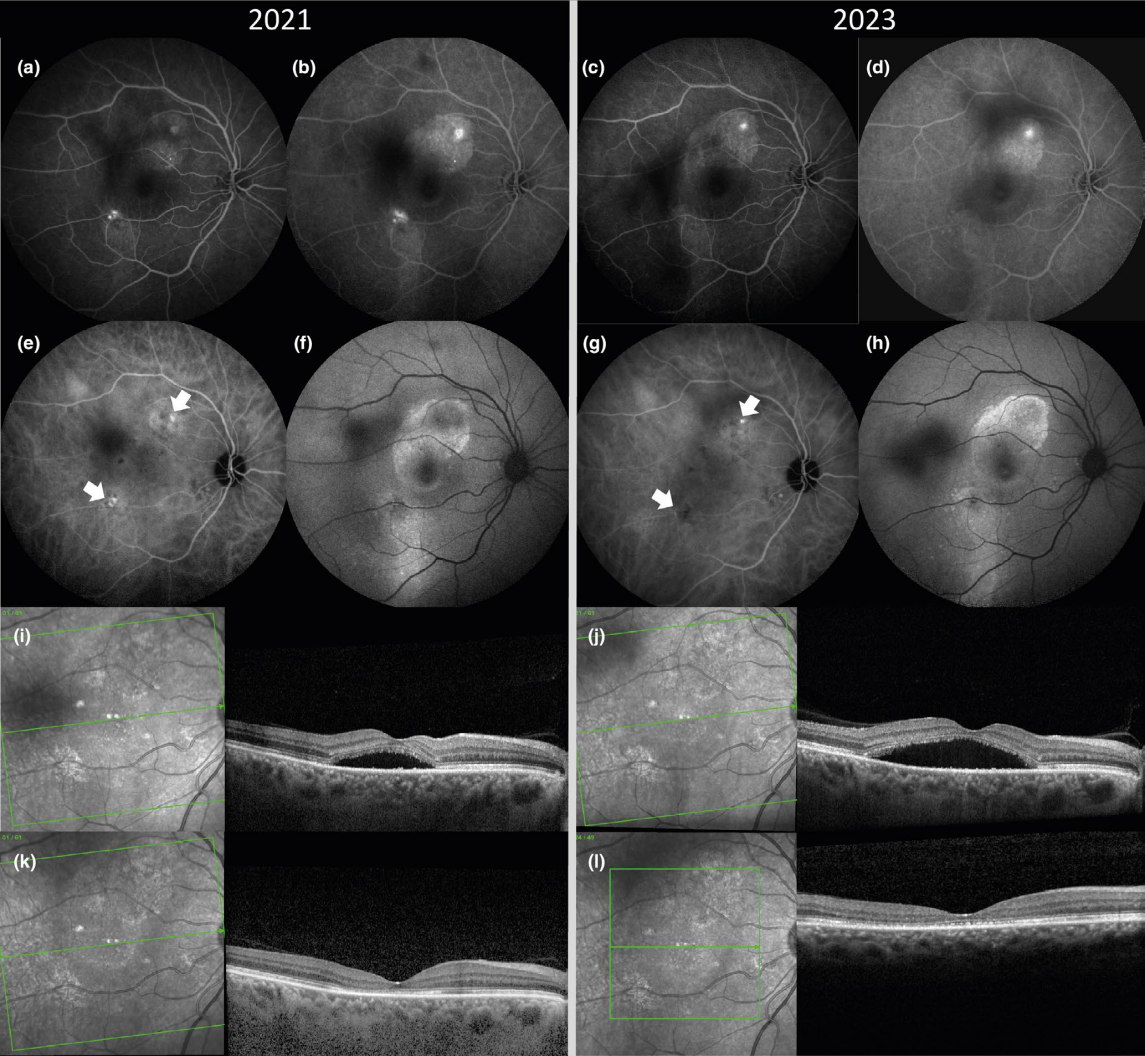
Multimodal imaging of a 61‐year‐old man with chronic central serous chorioretinopathy and vitreous floaters. Imaging was made before the first (a, b, e, f, i) and before the second photodynamic therapy (PDT) (c, d, g, h, j). The area of RPE changes did not change on fluorescein angiography (a–d) and fundus autofluorescence (f, h) between 2021 and 2023. Before the first PDT, there were two main areas of hypercyanescence on indocyanine green angiography (white arrows) (e). Both of these areas were treated with half‐dose PDT. The area of hypercyanescence was lower before the second PDT (white arrows) (g). Resolution of the subretinal fluid was observed after the first and after the second PDT (k, j).

The VA was comparable before the first and the second PDT, with a median VA of 0.15 LogMAR at both timepoints (*p* = 0.138), which increased to 0.05 LogMAR at the first follow‐up after both PDTs (*p* = 0.174). No differences were seen between the visits before and after both PDTs regarding CFT, ELM continuity and EZ continuity. On FA/FAF, the amount of DARA was higher before the second PDT (Table 2). The site of leakage on FA was different in 16/79 (20%) prior to the second PDT. At the second PDT, the area of hypercyanescence on ICGA was smaller in 14/33 (42%), equal in 15/33 (45%) and larger in 4/33 (12%). Figure 1 shows a patient with a decrease in hypercyanescence on ICGA between the first and the second episodes.

## 4 DISCUSSION

PDT with reduced settings is the mainstay treatment for non‐resolving CSC (Feenstra et al., 2024). After a successful PDT, a recurrence of SRF occurs in 6%–25% of patients (Fujita et al., 2015; Lai et al., 2015; Park et al., 2019; van Rijssen et al., 2021). It is currently unknown how CSC should be treated in the event of a recurrence of SRF after a previously effective PDT (with complete SRF resolution) for CSC in the same eye. In this multicentre retrospective study, we examined the safety and effectiveness of a second PDT upon the recurrence of SRF. After the second PDT, complete resolution of SRF occurred in 64% at the first follow‐up visit (median: 44 days [IQR 40–51]), the VA improved significantly and no increase in atrophy or newly formed atrophy was observed in any of the cases.

To study the effectiveness of a second PDT in recurrent CSC, we examined the complete resolution rate of SRF. Complete resolution was observed in 64% of patients at the first follow‐up and in 60% of patients at the second follow‐up after the second PDT. The percentage of resolution at the second follow‐up is most likely an underestimation, as there was a loss of follow‐up in eight patients with complete resolution at the first follow‐up. The resolution rate after the second PDT in this study is comparable to the effectiveness of PDT in treatment‐naive patients, as reported in two randomised controlled trials. In these trials, SRF resolution was observed in 51%–78% of patients after 1.5–3 months after half‐dose PDT (van Dijk et al., 2018; van Rijssen et al., 2022).

We studied the safety of a second PDT in terms of RPE atrophy, EZ continuity, CFT and VA, as PDT is known to cause both short‐ and long‐term choroidal changes. In the short‐term, hypoperfusion of the choriocapillaris is induced through damage to endothelial cells of the choriocapillaris, thrombosis and platelet activation. In the long‐term, decreased extravascular leakage, reduced width of dilated choroidal veins, and a decrease in choroidal thickness in regions within and outside the PDT laser spot are observed (Chan et al., 2003; Nassisi et al., 2017; Nishigori et al., 2023; Schlötzer‐Schrehardt et al., 2002). Nevertheless, these effects do not seem to cause RPE atrophy in the long term after PDT (Feenstra et al., 2023; Silva et al., 2013). In a previous report of bilateral CSC, a small number of eyes with multiple PDTs were included and RPE atrophy progression was not observed in these eyes (Pauleikhoff et al., 2023). In the current study, we did not observe RPE atrophy growth after the second PDT either. Additionally, we found improvement in the retina in terms of EZ continuity and CFT. The EZ was graded as continuous in 3% of patients before the second PDT and in 27% of patients at the first follow‐up after the second PDT. The CFT increased at follow‐up, which may possibly be explained by the recovery of the outer nuclear layer due to photoreceptor rearrangement and/or less mechanical stretching after SRF resolution (Torres‐Costa et al., 2021; van Rijssen et al., 2018). Furthermore, we evaluated safety based on VA. The median VA increased after the second PDT from 0.15 to 0.05 LogMAR (Table 2, Table S2). However, we observed a decline in VA with more than two lines after the second PDT in one patient. In this patient, subretinal fluid did not resolve directly after the PDT, which may partly explain the lack of VA improvement. There were no signs of progression of RPE atrophy on multimodal imaging in this patient and due to the loss of follow‐up, it is unclear if the patient's VA recovered over time. As we did not observe the progression of RPE atrophy in any patients and since a statistically significant increase in median VA was noticed, we conclude that a second PDT in recurrence of SRF in CSC seems to be a safe treatment option in the short term. Further studies, with a longer follow‐up period after the second PDT, are warranted to conclude whether performing a second PDT is also safe in the long term.

In addition to studying the safety and effectiveness of a second PDT, we also compared functional and anatomical characteristics between the first and the second PDT to study disease progression over a longer period of time. VA was comparable between both PDTs, as no difference was observed between the median VA before the first and the second PDT (0.15 and 0.15 LogMAR, respectively), nor in the median VA at follow‐up after the first and the second PDT (0.05 and 0.05 LogMAR, respectively). This is consistent with previous reports that showed long‐term preservation of vision after PDT (Feenstra et al., 2023; Silva et al., 2013). Moreover, we did not find significant anatomical differences between the first and the second PDT regarding CFT, ELM integrity and EZ integrity on OCT. An anatomical difference was observed in FA/FAF, in terms of an increase in DARA over time. Prior to the first PDT, DARA between 1 and 5 disc diameters was present in 35% of cases compared to 53% of cases after the second PDT (Table 2). The increase in DARA may be attributed to PDT, yet we rather assume that this increase is more likely caused by the recurrences of SRF and by choroidal dysfunction, which may itself lead to RPE alterations (Cheung et al., 2024; Gass, 1967). An anatomical difference was also observed at the site of leakage on FA, as the leakage point was at a different location in 20% of patients at the second PDT. As anatomical changes in multimodal imaging can evolve over time (Figure 1), and since the site of leakage may differ between the first and the second PDT, it is important to repeat multimodal imaging in case of recurrence of SRF.

This study has some limitations, the retrospective design of the study is one of them. Furthermore, 11% of patients had the second PDT performed within 6 weeks after the start of the CSC episode; we cannot rule out that some of these patients would have experienced spontaneous resolution of SRF. The second PDT was performed relatively early to prevent additional damage to the neuroretina and RPE that may arise from the cumulative presence of SRF. Moreover, no correction for multiple testing was applied for the compared characteristics between the first and the second PDTs; therefore, further studies are needed to validate these findings. Additionally, due to the reduced quality of the older OCT scan devices, it was not possible to assess all anatomical outcomes for every patient. However, this design allowed us to select a large number of patients with a relatively large follow‐up.

In conclusion, this study showed that performing a second PDT with reduced settings is safe and effective for recurrent CSC in the short term. Increased RPE alterations were observed prior to the second PDT, likely due to the cumulative presence of SRF. Therefore, a second PDT not only appears to be effective and safe, but may also be warranted to prevent further RPE changes and thus help preserve vision.

## FUNDING INFORMATION

This study was supported by Stichting Ooglijders (Rotterdam, the Netherlands); Stichting A.F. Deutman Oogheelkunde research fonds (Nijmegen, the Netherlands) and UitZicht project (2021‐15) (Delft, the Netherlands) that was financed by Algemene Nederlandse Vereniging ter Voorkoming van Blindheid; Stichting Blinden‐Penning; Landelijke Stichting voor Blinden en Slechtzienden (LSBS); Stichting Oogfonds Nederland; Rotterdamse Stichting Blindenbelangen; Stichting Oogfonds Nederland; Stichting Retina Nederland Fonds. The funding organizations had no role in the design or conduct of this research.

## Supporting information


Figure S1

Figure S2

Figure S3

Table S1

Table S2
