# Supplementary figures and images for: Effectiveness and safety of repeated photodynamic therapy in recurrent central serous chorioretinopathy
Source: Acta Ophthalmol. 2025 May 2;103(7):791–8. doi: 10.1111/aos.17511 (PMC12531609; doi:10.1111/aos.17511)

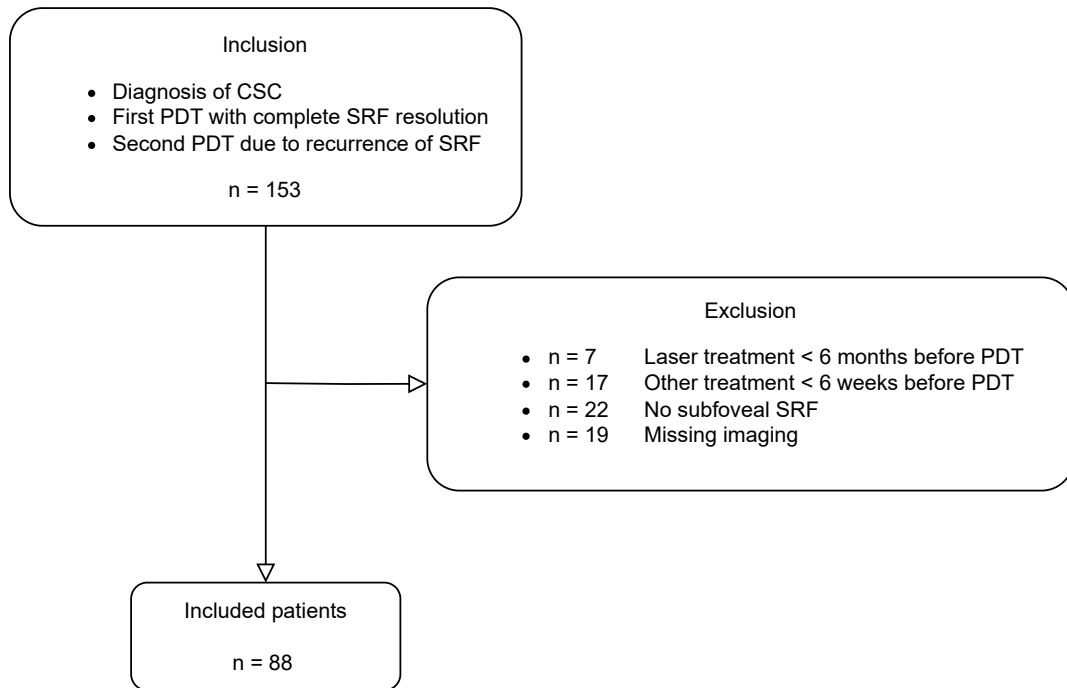

Supplement: Supplementary file 1 — Figure S1 [file AOS-103-791-s005.pdf]

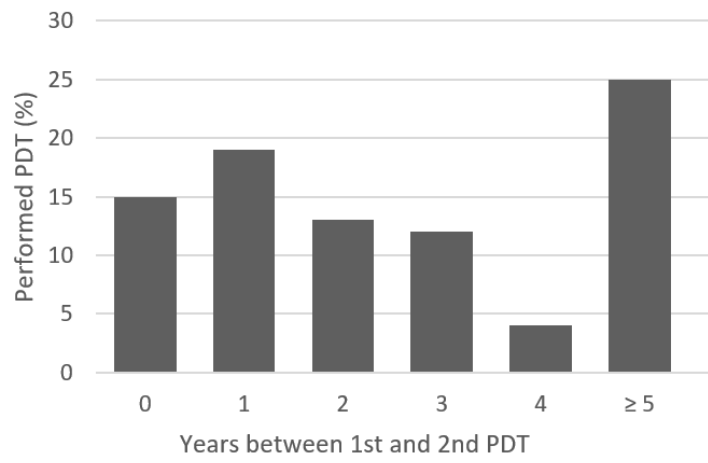

Supplement: Supplementary file 2 — Figure S2 [file AOS-103-791-s004.pdf]

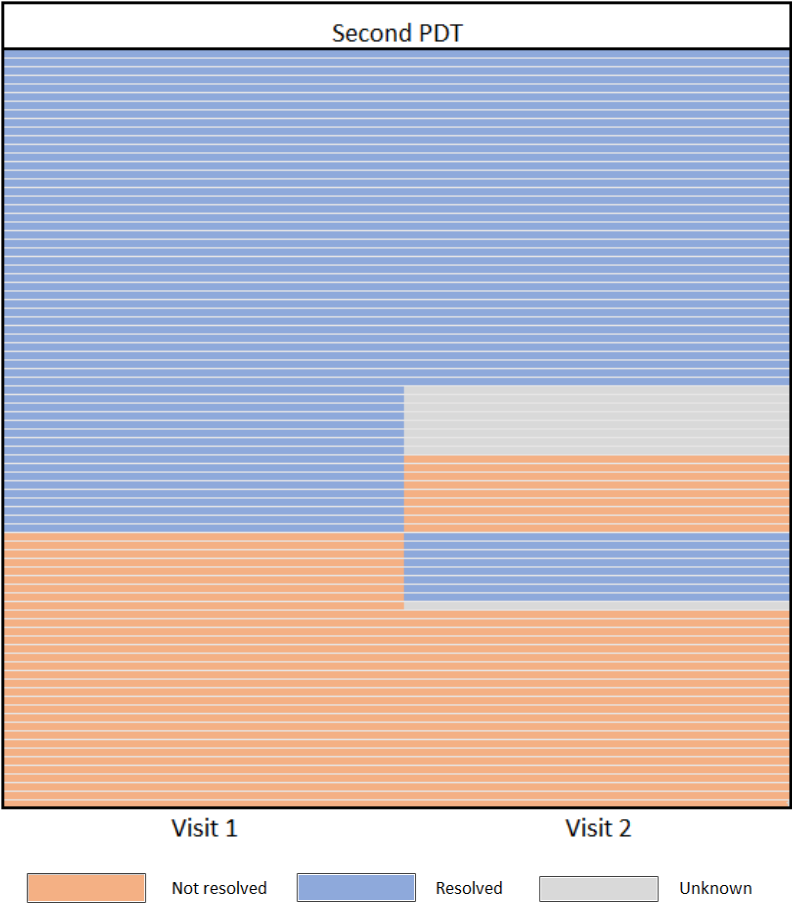

Supplement: Supplementary file 3 — Figure S3 [file AOS-103-791-s001.pdf]
