# Supplementary material for: Effectiveness and safety of repeated photodynamic therapy in recurrent central serous chorioretinopathy
Source: Acta Ophthalmol. 2025 May 2;103(7):791–8. doi: 10.1111/aos.17511 (PMC12531609; doi:10.1111/aos.17511)
Supplement: Supplementary file 4 — Table S1 [file AOS-103-791-s003.docx]

Table S1: Intergrader agreement of the outcomes that were measured/graded by two independent graders in patients with central serous chorioretinopathy.

|  | Intergrader agreement | 95% Confidence Interval |
| --- | --- | --- |
| ELM | 0.696* | 0.614 - 0.778 |
| EZ | 0.750* | 0.660 - 0.840 |
| CFT | 0.848† | 0.809 - 0.879 |
| DARA | 0.738* | 0.644 - 0.832 |
| FA leakage pattern | 0.752* | 0.648 - 0.856 |
| ICGA hyperpermeability pattern | 0.789* | 0.626 - 0.952 |
| † ICC of two-way mixed effects model with consistency definition. Average measures are reported.  * Cohen’s kappa  CFT, central foveal thickness; DARA, diffuse atrophic retinal pigment epithelium alterations, ELM, external limiting membrane; EZ, ellipsoid zone; FA, fluorescein angiography; ICC: Intraclass Correlation Coefficient; ICGA, indocyanine green angiography. | | |
