# Supplementary material for: Effectiveness and safety of repeated photodynamic therapy in recurrent central serous chorioretinopathy
Source: Acta Ophthalmol. 2025 May 2;103(7):791–8. doi: 10.1111/aos.17511 (PMC12531609; doi:10.1111/aos.17511)
Supplement: Supplementary file 5 — Table S2 [file AOS-103-791-s002.docx]

Table S2: Visual acuity and optical coherence tomography characteristics of patients with central serous chorioretinopathy at the first and second PDT. Characteristics of the visit before PDT, at the first follow-up, and at the second follow-up are presented.

| 1^st^ PDT | | | | | |
| --- | --- | --- | --- | --- | --- |
|  | Before PDT  (n=88) | First follow-up (n=88) |  | Second follow-up (n=73) |  |
| Visual acuity, Median [IQR], logMAR | 0.15 [0.05 - 0.30] | 0.05 [0.00 - 0.15] |  | 0.00 [0.00 - 0.13] |  |
| CFT, Mean (SD), μm | 112.5 ± 24.4 (n = 40) | 119.8 ± 22.9  (n = 44) |  | 121.8 ± 28.0  (n = 36) |  |
| ELM  Continuous  Interrupted | 13/40 (33%)  27/40 (68%) | 24/43 (56%)  19/43 (44%) |  | 20/33 (61%)  13/33 (39%) |  |
| EZ  Continuous  Interrupted | 0/40 (0%)  40/40 (100%) | 15/43 (35%)  28/43 (65%) |  | 10/33 (30%)  23/33 (69%) |  |
| 2^nd^ PDT | | | | | |
|  | Before PDT  (n=88) | First follow-up (n=88) | P-value (Before PDT – First follow-up) | Second follow-up (n=79) | P-value (Before PDT  – Second follow-up) |
| Visual acuity, Median [IQR], logMAR | 0.15 [0.05 - 0.30] | 0.05 [0.00 - 0.22] | < 0.001† | 0.05 [0.00 - 0.15] | < 0.001† |
| CFT, Mean (SD), μm | 105.5 ± 22.0  (n = 65) | 109.7 ± 24.1  (n = 65) | 0.043* | 115.9 ± 26.3  (n = 57) | < 0.001* |
| ELM  Continuous  Interrupted | 29/64 (45%)  35/64 (55%) | 40/64 (63%)  24/64 (38%) | 0.078‡ | 40/57 (70%)  17/57 (30%) | 0.017‡ |
| EZ  Continuous  Interrupted | 2/64 (3%)  62/64 (97%) | 17/64 (27%)  47/64 (73%) | < 0.001‡ | 21/57 (37%)  36/57 (63%) | < 0.001‡ |
| † Related samples Wilcoxon signed rank test  * Paired t-test (two-sided)  ‡ McNemar test  A P value of 0.05 was considered significant (P < 0.00625, after Bonferroni correction)  CFT, central retinal thickness; ELM, external limiting membrane; EZ, ellipsoid zone; FA, fluorescein angiography; FAF, fundus autofluorescence; LogMAR, logarithm of the minimum angle of resolution | | | | | |
